# Differences in pupillary light reflex between optic neuritis and ischemic optic neuropathy

**DOI:** 10.1371/journal.pone.0186741

**Published:** 2017-10-19

**Authors:** Yung Ju Yoo, Jeong-Min Hwang, Hee Kyung Yang

**Affiliations:** 1 Department of Ophthalmology, Kangwon National University Hospital, Kangwon National University Graduate School of Medicine, Chuncheon, Korea; 2 Department of Ophthalmology, Seoul National University College of Medicine, Seoul National University Bundang Hospital, Seongnam, Korea; Bascom Palmer Eye Institute, UNITED STATES

## Abstract

**Objectives:**

To determine the differences in pupillary light reflex (PLR) between the acute and chronic phases of optic neuritis (ON) and nonarteritic anterior ischemic optic neuropathy (NAION).

**Methods:**

The study included 30 patients with ON and 22 patients with NAION whose PLR were measured by a dynamic pupillometer (PLR-200; NeurOptics Inc., Irvine, USA). Age-matched controls included 58 healthy individuals with normal vision and optic nerve function. Pupil diameters, latency, constriction ratio, constriction velocity and dilation velocity were noted. The differences in PLR measurements were compared among the acute and chronic phases of ON and NAION, and in age-matched controls. Regression analysis determined factors associated with PLR measurements, including visual acuity, color vision defect, visual field defects and retinal nerve fiber layer thickness measurements on optical coherence tomography.

**Results:**

Pupillary constriction velocity, constriction ratio and latency were all significantly decreased in the acute phase of ON and NAION. ON showed significantly delayed constriction latency compared to NAION (P = 0.047). Pupillary constriction velocity, constriction ratio and latency were recovered in the chronic phase of ON (P = 0.038, 0.018, and 0.045), however, these parameters were not recovered in NAION (P = 0.693, 0.173 and 0.994).

**Conclusions:**

Pupillary constriction velocity, constriction ratio, and latency were significantly decreased in the acute phase of ON and NAION compared to normal controls. ON showed delayed constriction latency compared to NAION. Decreased PLR were recovered in the chronic phase of ON, but not in NAION.

## Introduction

Pupillary light reflex (PLR) is an objective marker reflecting optic nerve function [1]. Because PLR is related to the severity of optic nerve damage, evaluating PLR is a useful approach for neuro-ophthalmologic patients [2–4]. However, in practice, PLR is subjectively determined at a rough estimate, which depends much on the experience of the examiner, and the velocity and amplitude of the PLR cannot be easily quantified [5]. Nowadays, PLR can be monitored using various commercially available instruments [6–10]. Dynamic pupillometry offers the possibility for objective measurement and quantification of the PLR [11].

Optic neuritis (ON) and nonarteritic anterior ischemic optic neuropathy (NAION) are two of the most common causes of optic neuropathy in adults [12]. NAION is a sudden loss of vision related with decreased or interrupted blood flow to the affected optic nerve in older age groups [13]. Optic neuritis is a demyelinating inflammation of the optic nerve that typically occurs in young adulthood [14]. Optic disc edema can be present in both conditions, and features such as peripapillary hemorrhage may be useful for the differential diagnosis of optic neuropathy [15]. Occasionally, it is difficult to distinguish ON from NAION just by clinical manifestations such as the rate of onset, pattern of visual field loss, and optic disc appearance [16, 17]. Rizzo III et al.[16] reported that certain clinical factors such as pain and age distribution which had been thought to help differentiate between diseases could also overlap. As treatment strategies may differ according to the diagnosis, ancillary tests that could help distinguish between these etiologies would be useful in practical terms.

Optic nerve dysfunction leads to abnormal PLR in both ON and NAION [16, 18]. Although it is assumed that the pathophysiology of abnormal PLR in ON and NAION is different, there has been no study that revealed the distinguishable features of PLR between ON and NAION. In patients with severe visual loss due to optic neuropathy, characteristics of the afferent pupillary defect may differ according to the pathophysiology or stages of the disease [4]. Thus, quantifying the PLR can be helpful in the diagnosis and monitoring of patients with optic neuropathy.

In this study, we analyzed the different characteristics of PLR in the acute and chronic phases of ON and NAION, and evaluated the correlation of functional and structural parameters of the optic nerve with PLR characteristics in patients with different etiologies of optic neuropathy.

## Methods

This study adhered to the Declaration of Helsinki and the protocol was approved by the Institutional Review Board of Seoul National University Bundang Hospital (SNUBH) (IRB No.: SNUBH B-1604-344-115). All clinical investigation was conducted according to the principles expressed in the Declaration of Helsinki. Informed consent was not given, as patient records and information were anonymized and de-identified prior to analysis.

### Study subjects

We retrospectively analyzed patients who were diagnosed with ON or NAION between January 2011 and February 2016 in the Neuro-ophthalmology department of SNUBH. Diagnosis of ON and NAION was confirmed by two neuro-ophthalmologists (H.K.Y and J.M.H) and patients had no other pathological abnormalities on ocular examination. ON was defined as acute visual loss associated with ocular pain, abnormal color vision, and optic nerve inflammation confirmed by magnetic resonance image with enhancement [19]. NAION was clinically diagnosed based on acute painless visual loss, presence of optic disc edema, visual field defect, natural resolution of the disc edema within eight weeks and a normal erythrocyte sedimentation rate with age [20]. Patients with at least six months of follow-up after the onset of optic neuropathy were included. All patients in the ON group were treated with intravenous high dose steroid treatment in the acute phase and the NAION group was not treated with steroids. Patients performed dynamic pupillometry both in the acute phase and chronic phase. The definition of an acute phase was within six weeks from onset of visual acuity decrease and chronic phase was after six months of onset. In bilateral cases, when the disease developed at two different times, one eye was selected randomly for comparative analysis.

All patients underwent complete ophthalmic examination, including visual acuity assessment, automated refraction, slit lamp biomicroscopy, and dilated fundus examination. They also underwent measurements of the standard automated perimetry (Humphrey Field Analyzer program 30–2 full threshold; HVF, white stimulus, Carl Zeiss Meditec, Dublin, USA), and Goldmann perimetry (Haag-Streit, Bern, Switzerland) was used if the patient could not perform the HVF due to poor visual acuity defined as 20/80 or worse. Structural evaluation of the retinal nerve fiber layer (RNFL) was determined with the spectral-domain optical coherence tomography (Spectralis; Heidelberg Engineering, Heidelberg, Germany). Color vision tests (HRR; Richmond Hardy-Rand-Rittler Test) were also performed by one well-trained technician. Color vision deficiency was graded as mild, medium or severe, depending on whether or not the patient recognized the symbols on the higher contrast plates. For red-green color defects, patients who make errors in the two plates with the strongest contrast are graded as severe; those with an error in the three plates with medium contrast are graded as medium. For those who make errors within the five plates with mild contrast are graded as mild. Blue-yellow color defect was graded in a similar fashion.

Patients were excluded if they had any pathologic findings that might affect the PLR, such as glaucoma, vision affecting cataracts, and retinopathies. Patients with other conditions interfering with the PLR or their recordings such as congenital or acquired iris dysfunction, anterior segment abnormalities and severe ptosis were also excluded. Patients who were on medication known to affect the PLR, such as pilocarpine, atropine, selective serotonin reuptake inhibitors, and non-selective serotonin reuptake inhibitors were also excluded.

We selected an age-matched control group for both the ON group and NAION group from individuals with normal vision and no optic nerve dysfunction who had performed the dynamic pupillometry at the outpatient clinic of SNUBH. Fifty-eight healthy control subjects (mean age 45.74 ± 17.91 years) were included for comparison. The right eye was selected as a reference in controls.

### Pupillary light reflex measurements by dynamic infrared pupillometer

PLR were obtained and recorded by the same masked observer using the PLR-200 Pupillometer (NeurOptics Inc., Irvine, USA). PLR-200 is an automated monocular infrared pupillometer that records pupil images of each eye respectively. Pupillometry was performed after three minutes of dark adaption. To avoid pupil miosis caused by accommodation, we asked patients to fixate on a target at a distance of at least 3 meters away with the fellow eye. PLR-200 pupillometer has an eyecup designed for fitting the periorbital area which helps reduce the possibility of light entering the tested eye and standardize stimulus distance and intensity [21, 22]. Only one dim light source was used for fixation during measurements and darkness (< 0.3 lux) was confirmed via light meter measurement. Stimuli consisted of pulses of light with a fixed intensity of 180 Microwatts/cm^2^ and duration of 180 milliseconds. Visual light stimuli were presented using white light emitting diodes monocularly. Once the device has focused on the target pupil, a white light stimulus was flashed. Pupil size measurements were sampled at a frequency of 32-frames per second and lasted up to five seconds, allowing a full or partial recovery of the pupil size after light constriction. PLR of each eye was measured twice with an interval of 30 seconds and the average of data was used. PLR of each subject were measured in the right eye followed by the left. The device has been specifically designed to minimize possible inter-observer variability in evaluating pupillary reactions [23].

### Parameters of pupillary light reflex

As described in detail previously [24], eight PLR parameters were presented with pupil response curves [21]. The maximal pupil diameter (mm) was defined as the initial resting pupil size and minimal pupil diameter (mm) as the smallest pupil size during constriction. The pupillary constriction ratio (%) was defined as the difference between the maximum and minimum diameters divided by the maximal pupil diameter, and the latency (sec) as the time difference between initiation of retinal light stimulation and onset of pupillary constriction. Average constriction velocity (ACV, mm/sec) was defined as the amplitude of pupil constriction divided by the duration of constriction and average dilation velocity (ADV, mm/sec) as the amount of pupil size dilation after constriction divided by the duration of recovery to maximal pupil diameter. Maximal constriction velocity (MCV, mm/sec) was defined as the peak value of the velocity during constriction which is larger than the ACV. Total time from the peak of constriction to recovery of the pupil to 75% of maximal pupil diameter (T75, sec) was also measured.

### Statistical analysis

For statistical analysis, SPSS ver. 21.0 software (IBM Corporation, Armonk, NY, USA) was used. The comparison of PLR parameters and ocular characteristics between patients and age-matched controls, and between the patients with ON and NAION were performed using the independent *t*-test for continuous variables. Subgroup analysis of patients aged 35–70 years old was performed to exclude the effect of age on pupillary light reflex parameters. Repeatability of measurements in the normal fellow eye or controls was evaluated using the intraclass correlation coefficients (ICC). An ICC with its 95% confidence interval above 0.90 was considered to have a high level of repeatability and agreement. Linear regression was performed with pupillary constriction ratio and latency of PLR as the dependent variable to evaluate the relationship among several factors, including best corrected visual acuity, visual field mean deviation, average RNFL thickness in the circumpapillary region, mean temporal RNFL thickness, mean papillomacular bundle RNFL thickness, grade of red-green color vision defect and grade of blue-yellow color vision defect. Factors with a P-value of 0.05 or less in univariate analysis were included as candidate variables in the multivariate analysis. Paired *t*-test was conducted to determine if there was a statistically significant difference among the PLR of patients in the acute phase vs chronic phase. Subgroup analyses of PLR parameters were performed among patients (ON, NAION) and controls aged 35–70 years, and statistical analyses were performed using ANOVA with Bonferroni adjustment. According to Bonferroni-adjustment, results were considered statistically significant when the two-sided P-value was < 0.00625 (0.05/8 Bonferroni adjustment). Otherwise, a P-value < 0.05 was considered statistically significant. Data are presented as mean ± standard deviation.

## Results

### Demographics and ocular characteristics

Fifty-two patients diagnosed with ON (N = 30) and NAION (N = 22) were included and the mean age of onset was 38.3 ± 16.5 (range, 7.3–70.6 years) and 59.5 ± 11.6 years (range, 37.9–80.8 years), respectively (P = 0.001). The mean time of evaluation after onset for patients with ON and NAION were 7.5 ± 5.3 days and 18.5 ± 13.1 days, respectively (P < 0.001). The differences of ocular parameters between eyes with ON and NAION in the acute phase and age-matched controls are described in Table 1. ON and NAION patients had poor visual acuity (P < 0.001) and reduced visual field mean deviation (P < 0.001) compared to age-matched controls. Average RNFL thickness in the acute phase was significantly thicker in patients with NAION (P = 0.007) and ON (P < 0.001) compared to their age-matched controls.

Comparing patients with ON and NAION, there was no significant difference in visual acuity (P = 0.09) and visual field mean deviation (P = 0.08) between the two groups. Average, temporal and papillomacular bundle RNFL thickness in the acute phase showed no significant difference in patients with NAION compared to the ON group (P = 0.311, 0.067 and 0.085).

**Table 1:** Summary of pupil light reflex parameters and ocular characteristics between patients with optic neuritis and nonarteritic anterior ischemic optic neuropathy in the acute phase and controls.

	ON (N = 30)	Age-matched Control of ON (N = 36)	*P Value*	NAION (N = 22)	Age-matched Control of NAION (N = 22)	*P Value*
M:F (N)	10:20	16:20	0.361*	13:9	10:12	0.371*
Laterality (R:L)	14:16	36:0		12:10	22:0	
Symptom duration (day)	7.5±5.3 (1,20)			18.5±13.1 (1,43)		
Age (year)	38.3±16.5 (7.3,70.6)	40.0±16.9 (7.3,69.1)	0.675	59.5±11.6 (37.9,80.8)	60.0±11.7(36.9,78.3)	0.888
Visual acuity (LogMAR)	1.08±0.75 (-0.17,2.00)	0.01±0.05 (-0.10,0.08)	**<0.001**	0.93±0.61 (0.10,2.00)	-0.08±0.19 (-0.50,0.18)	**<0.001**
Visual field MD (dB)	-10.01±8.51 (-28.68,-1.45)	-0.95±1.48 (-4.19,1.27)	**0.008**	-16.07±6.51 (-26.58,-4.68)	-1.35±1.45 (-5.12,0.52)	**<0.001**
Average RNFL thickness (μm)	139.3±43.8 (71,238)	102.6±7.6 (85,116)	**<0.001**	156.8±70.0 (70,283)	103.1±7.0 (86,111)	**0.007**
Temporal RNFL thickness (μm)	94.2±24.7 (56,162)	79.9±12.8 (64,117)	**0.009**	126.5±75.8 (41,251)	77.7±21.0 (57,131)	0.171
PMB RNFL thickness (μm)	64.6±12.5 (43,87)	59.0±9.1 (43,80)	0.077	102.3±78.1 (31,286)	54.3±13.7 (39,90)	0.164
Red-green color defect (N)^†^	4:6:2:0:18	36:0:0:0:0	**<0.001**	10:6:0:0:6	22:0:0:0:0	**<0.001**
Blue-yellow color defect (N)^†^	2:6:3:1:18	36:0:0:0:0	**<0.001**	8:5:0:3:6	22:0:0:0:0	**<0.001**
Maximal pupil diameter (mm)	5.5±0.7 (4.0,7.2)	5.7±0.9 (3.7,7.2)	0.241	5.1±1.0 (2.9,6.5)	5.4±1.0 (3.6,6.8)	0.155
Minimal pupil diameter (mm)	4.4±0.8 (3.0,6.2)	3.9±0.7 (2.4,5.2)	0.019	3.9±1.0 (2.1,5.5)	3.7±0.8 (2.3,5.1)	1.000
Pupil constriction ratio (%)	19.8±10.4 (1,36)	31.4±3.4 (26,41)	**<0.001**	23.8±7.6 (10,34)	32.4±3.5 (26,40)	**<0.001**
Latency (sec)	0.28±0.06 (0.19,0.43)	0.23±0.02 (0.19,0.25)	**<0.001**	0.26±0.02 (0.22,0.28)	0.23±0.02 (0.19,0.25)	**<0.001**
ACV (mm/s)	2.56±1.18 (0.10,4.40)	3.77±0.43 (2.54,4.47)	**<0.001**	2.76±0.73 (1.75,3.99)	3.69±0.46 (2.73,4.49)	**<0.001**
MCV (mm/s)	3.33±1.55 (0.38,5.03)	4.87±0.52 (3.41,5.84)	**<0.001**	3.32±1.18 (0.67,5.05)	4.75±0.54 (3.54,5.51)	**<0.001**
ADV (mm/s)	0.72±0.28 (0.20,1.20)	0.92±0.15 (0.60,1.19)	**0.001**	0.74±0.22 (0.39,1.29)	0.95±0.19 (0.50,1.27)	**0.002**
T75 (sec)	1.63±0.80 (0.97,3.55)	1.92±0.63 (1.02,2.00)	**0.001**	1.73±0.66 (0.83,3.52)	1.35±1.45 (0.52,5.12)	0.352

ACV, Average constriction velocity; ADV, Average dilation velocity; MD, mean deviation; NAION, nonarteritic ischemic optic neuropathy; ON, optic neuritis; PMB, papillomacular bundle; RNFL, retinal nerve fiber layer; T75, Total time from the peak of constriction to recovery of the pupil to 75% of maximal pupil diameter

Continuous variables are given as mean with standard deviations (range).

P values by independent *t-*test for continuous variables.

*P value by Chi-square test.

Factors with statistical significance are shown in boldface.

^†^The number of patients showing the following results by the HRR color vision test: ‘Normal:Mild:Moderate:Severe:Demonstration failure’.

### Pupillary light reflex parameters

PLR-200 dynamic pupillometer showed a high level of repeatability (ICC > 0.9) for intra-individual maximal and minimal pupil diameter, constriction ratio, ACV, and MCV and a moderate level of repeatability (ICC > 0.8) for constriction latency, ADV and T75. Comparisons of mean values of the eight PLR parameters between patients and controls are presented in Table 1. Maximal pupil diameter revealed no significant difference between patients and controls (P = 0.241 for the ON group, P = 0.155 for the NAION group). Pupillary constriction ratio was significantly decreased in patients with ON and NAION (mean; 19.8% and 23.8% respectively) compared to controls (mean; 31.7%, P < 0.001). In addition, constriction latency was significantly delayed in patients with ON and NAION (mean; 0.28 and 0.26 sec, respectively) compared to controls (mean; 0.23 sec, P < 0.001). ACV, MCV and ACV were all significantly decreased in patients with ON and NAION compared to their age-matched controls (P<0.001).

**Fig 1:**
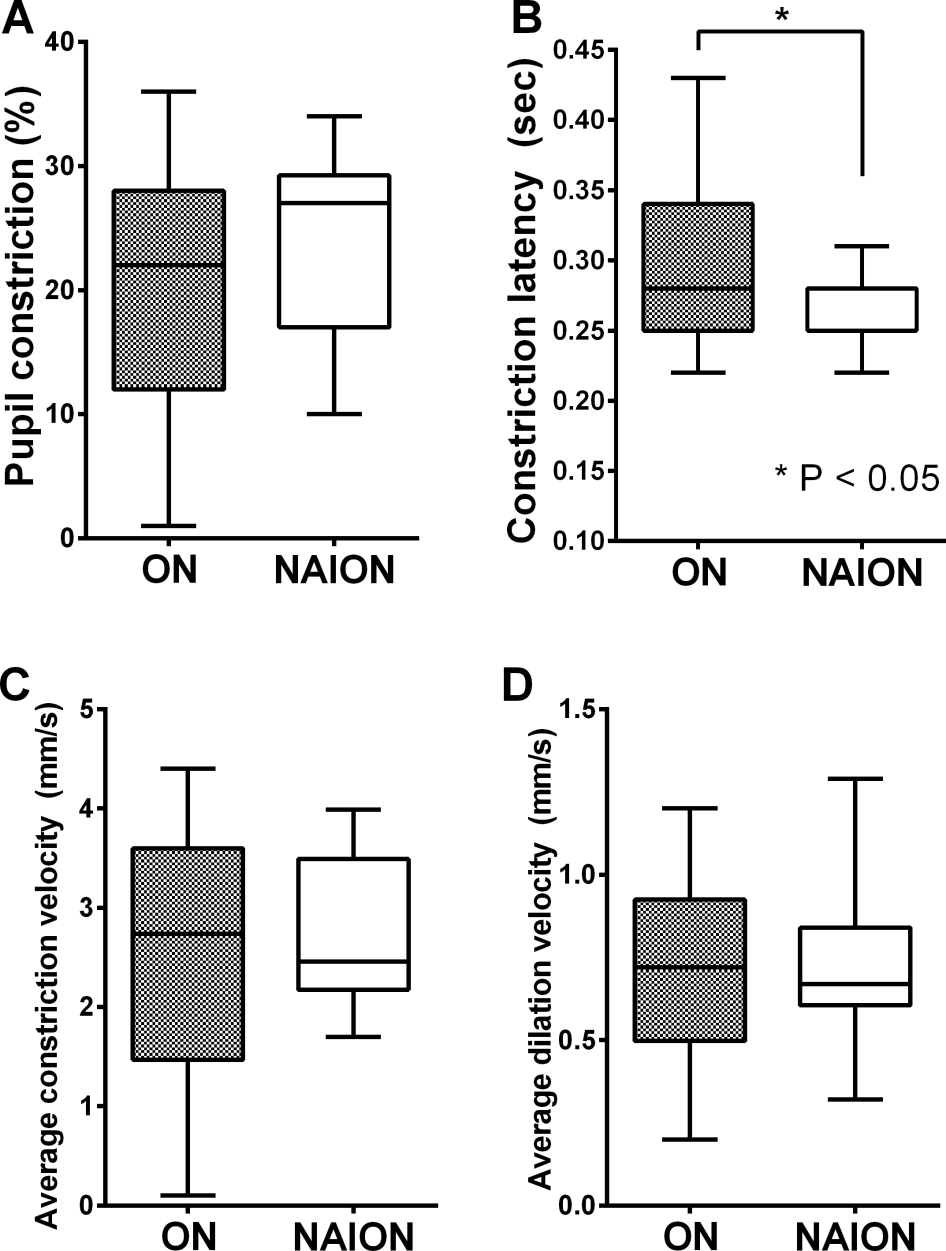
**Comparison of pupillary constriction ratio (A), constriction latency (B), average constriction velocity (C), and average dilation velocity (D) between patients with optic neuritis (ON) and nonarteritic ischemic optic neuropathy (NAION).** (A, C, D) Pupillary constriction ratio, average constriction velocity, and average dilation velocity revealed no significant difference between the ON group and NAION group. (P values = 0.069, 0.585 and 0.869, respectively). (B) However, constriction latency was significantly delayed in patients with ON compared to NAION (P = 0.047).

Constriction latency was more delayed in patients with ON compared to patients with NAION as shown in Fig 1 (P = 0.047). However, constriction ratio, ACV, and ADV did not show significant inter-group differences (P values for the constriction ratio, ACV, and ADV = 0.069, 0.585 and 0.869).

In the subgroup analysis of patients aged 35–70 years old with ON (19 patients) and NAION (16 patients), there was no significant difference in visual acuity (LogMAR 1.02 ± 0.76 in the ON group and 0.80 ± 0.65 in the NAION group) and visual field mean deviation (-10.42 ± 9.72 dB in the ON group and -16.1 ± 5.4 dB in the NAION group) between the two groups (P = 0.105 and 0.115, respectively). Average, temporal and papillomacular bundle RNFL thickness in the acute phase showed no significant difference in patients with NAION compared to the ON group (P = 0.898, 0.182, 0.185). Pupillary constriction ratio, constriction latency, ACV, MCV and ADV were all significantly decreased in patients with ON and NAION compared to age-matched controls (P < 0.001). Constriction latency was more delayed in patients with ON compared to NAION (0.29 ± 0.06 and 0.26 ± 0.02 sec, respectively) (P = 0.024). However, constriction ratio, ACV, MCV, and ADV did not show significant inter-group differences (P = 0.05, 0.118, 0.453 and 0.292, respectively).

In the subgroup analysis of patients with unilateral ON (19 patients) and NAION (12 patients), pupillary constriction ratio, ACV, MCV, and ADV showed significant correlation with the clinical grading of RAPD in both unilateral ON and NAION (P = 0.001, 0.131, 0.001, and 0.001 respectively).

### Comparison between ON and NAION in acute and chronic phases

**Fig 2:**
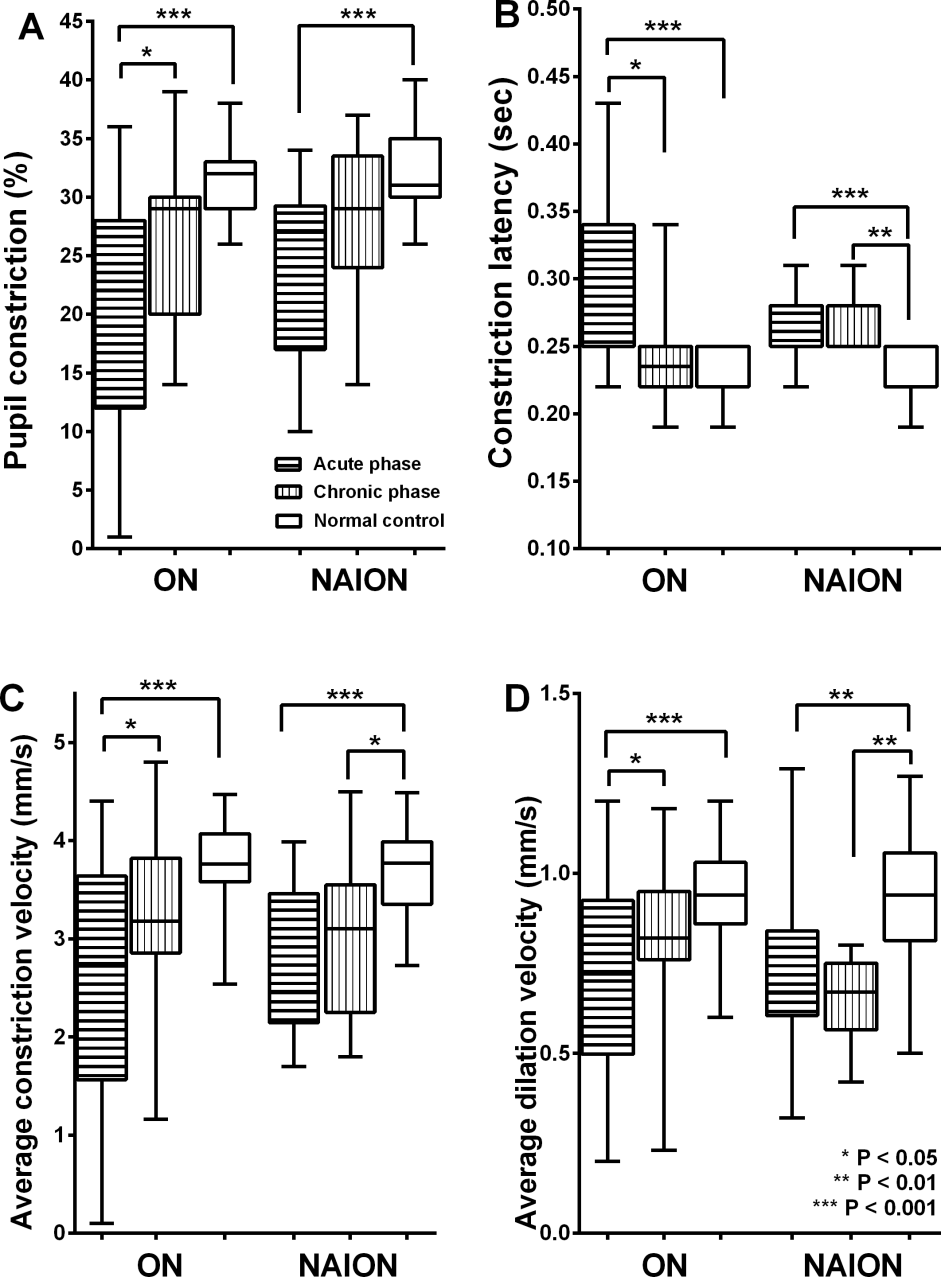
**Comparison of pupillary constriction ratio (A), constriction latency (B), average constriction velocity (ACV) (C), and average dilation velocity (ADV) (D) among patients with acute and chronic phases of optic neuritis (ON), nonarteritic ischemic optic neuropathy (NAION) and normal controls.** In the ON group, all these four PLR parameters improved in the chronic phase compared to those in the acute phase (all P < 0.05). On the contrary, there were no significant differences of four parameters between acute and chronic phases in the NAION group.

Comparisons of the four PLR parameters among acute and chronic phases of patients with ON and NAION and controls are presented in Fig 2. In the chronic phase, there was no significant difference in LogMAR visual acuities (0.22 ± 0.27 in the ON group and 0.48 ± 0.43 in the NAION group) and visual field mean deviation (-3.2 ± 2.3 dB in the ON group and -4.5 ± 2.7 dB in the NAION group) between the two groups (P = 0.585 and 0.263, respectively). Maximal pupil diameter was also not significantly different among patients in the acute and chronic phases.

In the chronic phase of ON, pupillary constriction ratio significantly improved compared to the acute phase (26.5 ± 9.2% vs 20.3 ± 10.7%, P = 0.018) and constriction latency was shorter than that in the acute phase (0.24 ± 0.06 vs 0.27 ± 0.04 sec, P = 0.045). On the contrary, in the chronic phase of NAION, pupillary constriction ratio (28.2 ± 6.9 vs. 23.8 ± 7.6%, P = 0.173) and latency (0.26 ± 0.02 vs. 0.26 ± 0.03 sec, P = 0.994) was similar to that in the acute phase. In the chronic phase, the ON group had improved in ACV (P = 0.042), MCV (P = 0.025) and ADV (P = 0.038) compared to the acute phase. These chronic phase parameters were not significantly different compared to controls (P values for ACV, MCV, ADV = 0.191, 0.310 and 0.961, respectively). On the other hand, patients with NAION revealed no significant improvement of ACV, MCV, and ADV in the chronic phase (P values for ACV, MCV, and ADV = 0.693, 0.183, and 0.512, respectively).

### Factors associated with abnormal pupillary light reflex

Tables 2 and 3 show the results of logistic regression analysis to assess factors associated with the degree of abnormal PLR in patients with ON and NAION.

**Table 2:** Factors associated with pupillary constriction ratio in the acute phase.

	ON					NAION			
		Univariate analysis		Multivariate analysis 1		Multivariate analysis 2		Univariate analysis	
		ß ± SE	P value	ß ± SE	P value	ß ± SE	P value	ß ± SE	P value
VA (LogMAR)		5.054 ± 1.712	**0.006**	13.763 ± 5.316	**0.049**	-1.938 ± 5.047	0.717	-0.18±2.85	0.952
VF MD (dB)		0.701 ± 0.180	**0.005**			0.511 ± 0.638	0.460	-0.11±0.29	0.712
Mean RNFL thickness (μm)		-0.008 ± 0.048	0.868					-0.02±0.03	0.444
Temporal RNFL thickness (μm)		0.011 ± 0.021	0.622					0.01±0.02	0.760
PMB RNFL thickness (μm)		0.069 ± 0.191	0.722					0.04±0.02	**0.050**
RG defect		-2.906 ± 1.007	**0.007**	-10.186 ± 3.028	**0.020**			-0.02±1.01	0.983
BY defect		-3.467 ± 1.161	**0.006**			1.338 ± 2.470	0.611	0.07±1.01	0.944

BY defect, blue-yellow color vision defect tested by the HRR color vision test; NAION, nonarteritic ischemic optic neuropathy; ON, optic neuritis; PMB, papillomacular bundle; RG defect, red-green color vision defect tested by the HRR color vision test; RNFL, retinal nerve fiber layer; SE, standard errors; T75, Total time from the peak of constriction to the recovery of pupil size to 75% of maximal pupil diameter; VA, visual acuity; VF MD, visual field mean deviation.

Factors with statistical significance are shown in boldface.

**Table 3:** Factors associated with pupillary constriction latency in the acute phase.

	ON				NAION			
	Univariate analysis		Multivariate analysis 1		Multivariate analysis 2		Univariate analysis	
	ß ± SE	P value	ß ± SE	P value	ß ± SE	P value	ß ± SE	P value
VA (LogMAR)	0.029 ± 0.010	**0.005**					0.08 ± 0.11	0.992
VF MD (dB)	-0.002 ± 0.001	**0.005**	-0.001 ± 0.001	0.084	-0.001 ± 0.001	0.084	-0.02 ± 0.01	0.369
RNFL mean thickness (μm)	0.001 ± 0.001	0.624					0.00 ± 0.00	0.557
Temporal RNFL thickness (μm)	0.001 ± 0.001	0.741					0.00 ± 0.01	0.631
PMB RNFL thickness (μm)	0.001 ± 0.001	0.859					0.00 ± 0.01	0.557
RG defect	0.015 ± 0.006	**0.021**					0.001 ± 0.003	0.636
BY defect	0.018 ± 0.007	**0.017**					0.002 ± 0.003	0.540

BY defect, blue-yellow color vision defect tested by the HRR color vision test; NAION, nonarteritic ischemic optic neuropathy; ON, optic neuritis; PMB, papillomacular bundle; RG defect, red-green color vision defect tested by the HRR color vision test; RNFL, retinal nerve fiber layer; SE, standard errors; T75, Total time from the peak of the constriction to the recovery of the pupil to 75% of maximal pupil diameter; VA, visual acuity; VF MD, visual field mean deviation.

Factors with statistical significance are shown in boldface.

In the ON group, worse visual acuity (P = 0.006), worse visual field mean deviation (P = 0.005), and worse red-green and blue-yellow color vision defect were significantly associated with the degree of abnormal pupillary constriction ratio in univariate analysis. Multivariate analysis was performed to account for the collinearity between the red-green color vision defect and blue-yellow color vision defect. Kendall's rank correlation coefficient was significant (0.864, P < 0.001). Multivariate analysis revealed that red-green color vision defect (P = 0.020) and visual acuity (P = 0.049) were associated with the severity of abnormal pupillary constriction ratio. However, only worse visual field mean deviation showed borderline significance in relation with the degree of abnormal latency in multivariate analysis (P = 0.084).

In the NAION group, a thicker mean papillomacular bundle RNFL thickness in the acute phase showed borderline significance (P = 0.05) in relation with pupillary constriction ratio (%) by univariate analysis, however, this was not significant in multivariate analysis. There was no correlation between ocular characteristics and constriction latency (all P > 0.05).

## Discussion

PLR is usually examined subjectively, using a pen flashlight and a pupil gauge in the clinical setting. These methods are affected by the examiner's experience and subject to inter-examiner variability [5]. Our study objectively measured the PLR and showed marked decrease of pupillary constriction ratio and delayed latency in the acute phase of ON and NAION. In comparison of the two groups, ON showed more delayed latency compared to NAION. On the other hand, in the chronic phase, patients in the ON group showed recovery of pupillary constriction ratio and latency to normal levels while these parameters were not recovered in the NAION group.

ON is the most frequent optic neuropathy encountered in general ophthalmic practice which results from inflammation of the optic nerve from any cause, yet by convention, it has come to mean optic nerve damage due to demyelination [25]. NAION represents an optic nerve disorder which results from insufficient perfusion to the optic nerve head. Axoplasmic stasis and swelling arise from decreased perfusion and are followed by axonal dropout and loss of retinal ganglion cells [26].

In this study, we analyzed the PLR of patients diagnosed with ON and NAION compared to age-matched controls using an automated and objective dynamic pupillometer. We revealed that in the acute phase of ON and NAION, the PLR was significantly reduced in the affected eye compared with normal controls. The pathophysiology of optic nerve dysfunction in the acute phase can be explained by active inflammation in the ON group and ischemic damage in the NAION group. In addition, constriction latency is more prolonged in ON compared to NAION. There is no previous study that directly compared the difference in pupillary constriction latency between the two groups. We postulated that the reason for this difference in constriction latency between ON and NAION is the conduction delay prominent in ON. Demyelination occurs early in ON and is associated with acute inflammation and macrophage ingestion of myelin [21]. Demyelination in ON may include the entire thickness of the optic nerve and up to several centimeters in distribution [27]. On the contrary, NAION results from a sudden ischemic insult to the proximal portion of optic nerve which is initially confined to the optic nerve head [13]. This pathophysiologic difference also made contrasting features of visual evoked potential between the two groups. NAION showed reduction in visual evoked potential amplitude, often severe, with little or no change in latency [28], unlike the marked delay in latency that occurred in ON [27].

Another important finding in our study is that despite the structural change of RNFL thinning in the chronic phase of both ON and NAION, only the NAION group showed significant reduction of PLR and delayed latency in the chronic phase, while the ON group showed improvement of these parameters. In our study, all patients with ON were treated with intravenous high dose steroid in the acute phase while patients in the NAION group were not treated with steroids. In the Optic Neuritis Treatment Trial study, visual recovery of demyelinated ON occurred rapidly in most patients [29] and continued for up to a year in the affected eye; the median visual acuity in the affected eye at one year after the onset of ON was 20/16, with more than 90% of patients having a visual acuity of better than 20/40 [29]. A previous study revealed that an ongoing process of remyelination and reorganization of ion channel distribution help to restore conduction in a demyelinated axon of ON [30]. Reorganization of ion channel distribution is capable of restoring conduction along the demyelinated nerve within a few weeks [31]. This may contribute to the recovery of symptoms after an acute episode of demyelination and can also explain the recovery of PLR in the chronic phase of ON in our study.

In our study, pupillary constriction ratio of patients with NAION was significantly associated with mean PMB RNFL thickness. Because the damage of PLR was related with the amount of axonal damage, our results are consistent with those of Bellusci et al. [32] who reported that the location of RNFL changes in the acute phase matched with that of visual field loss. On the contrary, in the ON group, decreased pupillary constriction ratio of patients showed no significant association with RNFL thickness. Kupersmith et al. [24] found that RNFL thickening can also occur when the demyelinating lesion is remote from the globe and do not seem to be associated with increased severity of visual loss at presentation or poor recovery of vision in ON. Another study revealed that if the affected eye had no clinically evident optic disc swelling at the time of presentation in ON, there was no significant increase in the mean RNFL thickness compared with the unaffected fellow eye [33]. This is in line with our study that there is no significant association between decreased pupillary constriction ratio and mean RNFL thickness in the ON group. On the other hand, pupillary constriction ratio was significantly related to decreased visual acuity and red-green color vision defect in the ON group. Visual acuity decrease and dyschromatopsia are typical features of acute ON that recover after time [29]. Our study showed that the amount of pupillary dysfunction was closely related with visual dysfunction, that were both reversible in ON.

There was no significant correlation between pupillary constriction latency and initial ocular parameters in the NAION group (all P > 0.1). The pupillary constriction latency may reflect delay in afferent visual processing, which is less dependent on the efferent pathway of the light reflex compared to amplitude measurements. Previous studies have shown that constriction latency can be delayed in patients with demyelinating disease [34], Leber’s hereditary optic neuropathy [35], amblyopia [36], and optic atrophy. The accuracy and resolution of constriction latency measurements were improved by higher sampling rates, and the absolute value of latency became shorter with higher light intensity [37]. Because we used a sampling rate of 32 Hz and recording was repeated only twice, there might be a limitation on the reliability of our data. However, as compared to other monocular pupillometers, the NeurOptics PLR-200 pupillometer demonstrated the best inter-observer agreement and higher inter- and intra-observer repeatability [38]. The reliability and robustness of pupil tracking algorithms and the corresponding precision and accuracy in pupil measurements have been evaluated and described [39–42]. Further analysis using pupillometry with higher frequencies or multiple measurements can improve our understanding in the association between constriction latency and other ocular parameters.

Our study has several limitations. First, the age disparity between ON and NAION groups may affect the results. Sharma et al.[43] evaluated factors influencing the PLR in healthy Asian individuals and reported that aging is associated with reduction of PLR at specific light stimuli. Another previous report using a video pupillometer has shown a significantly smaller base pupil diameter and MCV in the older group, suggesting that a smaller pupil diameter might underestimate the pupillary constriction ratio [44]. Thus, we performed a subgroup analysis (not shown) in patients over fifty and compared ON with NAION in these patients. However, in this subgroup of patients, there was no difference in the results compared to our present study. Moreover, there was no statistically significant difference in PLR parameters between the two age-matched controls (young and old) of each group. Third, in the case of chronic phase results, the mean deviation of visual field tests may have been overestimated because it was performed only in patients with good visual acuity. Fourth, all patients with ON were treated with intravenous high dose steroid. Therefore, the results of our study do not reveal how the PLRs of untreated ON patients would change in the chronic phase. Finally, the maximal interval of onset to presentation did not exceed three weeks in the ON group, but some patients with NAION came to the neuro-ophthalmology clinic after three weeks. Because NAION showed slower recovery compared with ON, we included patients who visited our clinic up to six weeks from symptom onset as the acute phase of NAION.

In conclusion, pupillary constriction velocity, constriction ratio, and latency were significantly decreased in the acute phase of ON and NAION compared to controls. Constriction latency was more delayed in patients with ON than NAION in the acute phase. In addition, decreased constriction velocity and constriction ratio, and delayed latency were recovered in the chronic phase of ON, but not in NAION. Identifying the deterioration of specific PLR parameters in the acute phase and recovery in the chronic phase help understand the underlying pathophysiology of various optic neuropathies. Dynamic pupillometry is an objective method that can reveal the different characteristics of PLR in ON and NAION.
